# NLRP3 activation in neutrophils induces lethal autoinflammation, liver inflammation, and fibrosis

**DOI:** 10.1038/s44319-023-00021-5

**Published:** 2024-01-12

**Authors:** Benedikt Kaufmann, Aleksandra Leszczynska, Agustina Reca, Laela M Booshehri, Janset Onyuru, ZheHao Tan, Alexander Wree, Helmut Friess, Daniel Hartmann, Bettina Papouchado, Lori Broderick, Hal M Hoffman, Ben A Croker, Yanfang Peipei Zhu, Ariel E Feldstein

**Affiliations:** 1 Department of Pediatrics, University of California San Diego, La Jolla, CA USA; 2 Department of Surgery, TUM School of Medicine, Klinikum rechts der Isar, Technical, University of Munich, Munich, Germany; 3 Department of Hepatology and Gastroenterology, Charité, Universitätsmedizin Berlin, Berlin, Germany; 4 Department of Pathology, University of California San Diego, La Jolla, CA USA

**Addendum to**: *EMBO Reports* (2022) 23:e54446. 10.15252/embr.202154446 | Published online 4 October 2022

In this article, the ‘Disclosure and competing interests statement’ section is incomplete.

(Addendum text in bold)

Disclosure and competing interests statement

L.B. is a site PI for Novartis, Inc. H.H. is a consultant for Novartis. H.H. has research collaborations with Jecure, Inc., Zomagen, Inc and Takeda, Inc. A.F. is a consultant for Ventyx Bio, Inc, Novo Nordisk, Inipharm. A.F. has research collaborations with Takeda, Inc. **A.F is an employee and stockholder of Novo Nordisk (Since 07/2022)**.

This change does not affect the text or interpretation of the article.

